# Maternal mortality in Sweden 1988–2007: more deaths than officially reported

**DOI:** 10.1111/aogs.12037

**Published:** 2012-12-04

**Authors:** Annika Esscher, Ulf Högberg, Bengt Haglund, Birgitta Essën

**Affiliations:** Department of Women's and Children's Health, International Maternal and Child Health (IMCH), Uppsala UniversityUppsala, Sweden

**Keywords:** Maternal mortality, pregnancy-related mortality, underreporting, Sweden, vital statistics, ICD

## Abstract

**Objective:**

To obtain more accurate calculations of maternal and pregnancy-related mortality ratios in Sweden from 1988 to 2007 by using information from national registers and death certificates.

**Design:**

A national register-based study, supplemented by a review of death certificates.

**Setting:**

Sweden, 1988–2007.

**Population:**

The deaths of 27 957 women of reproductive age (15–49 years).

**Methods:**

The Swedish Cause of Death Register, Medical Birth Register, and National Patient Register were linked. All women with a diagnosis related to pregnancy in at least one of these registers within 1 year prior to death were identified. Death certificates were reviewed to ascertain maternal deaths. Maternal mortality ratio (the number of maternal deaths/100 000 live births, excluding and including suicides), and pregnancy-related mortality ratio (number of deaths within 42 days after termination of pregnancy, irrespective of cause of death/100 000 live births) were calculated.

**Main outcome measures:**

Direct and indirect maternal deaths and pregnancy-related deaths.

**Results:**

The maternal mortality ratio in Sweden, based on the current method of identifying maternal deaths, was 3.6. After linking registers and reviewing death certificates, we identified 64% more maternal deaths, resulting in a ratio of 6.0 (or 6.5 if suicides are included). The pregnancy-related mortality ratio was 7.3. A total of 478 women died within a year after being recorded with a diagnosis related to pregnancy.

**Conclusions:**

By including the 123 cases of maternal death identified in this study, the mean maternal mortality ratio from 1988 to 2007 was 64% higher than reported to the World Health Organization.

**Key Message**

Accurate surveillance of maternal mortality, even in countries with few cases like Sweden, requires better tools. More complete reporting can be achieved by using information available from national registers and death certificates.

## Introduction

Although 99% of maternal deaths occur in low-income countries, healthy young women still die in high-income countries due to complications of pregnancy and childbirth. Maternal mortality decreased in Europe until the 1980s 1–3 but recent data from several European countries have indicated increasing maternal mortality rates 4–7. In part, these changes are due to improved data assessment, but demographic changes such as increased maternal age and migration may be contributing to this rise 4,5,8–10. Underestimation of maternal mortality appears to be substantial, even in high-income countries 11–13, including Sweden 15,16. Inadequate statistics not only impede the possibility of determining trends but also affect inter-country comparisons of maternal mortality rates 11,17. Although the absolute number is small, maternal mortality is tragic, largely because it is often preventable. Accurate surveillance of maternal deaths may therefore lead to changes in patient care.

The World Health Organization (WHO) defines maternal death in International Classification of Diseases (ICD)-9 and ICD-10 2004 as the death of a woman while pregnant or within 42 days (that is, days 0–41) of termination of pregnancy – irrespective of the duration and site of the pregnancy – from any cause related to or aggravated by the pregnancy or its management, but not from accidental or incidental causes. This definition allows the identification of maternal deaths, based on their causes, as either direct or indirect. Direct deaths are those resulting from complications to pregnancy, delivery, and postpartum; from interventions, omissions or incorrect treatment; or from a chain of events resulting from any of the above. Indirect deaths are those resulting from preexisting disease or diseases that developed during pregnancy but were not due to direct obstetric causes, although they may have been aggravated by physiological effects of pregnancy. Deaths due to hemorrhage, puerperal sepsis or complications of anesthesia are examples of direct maternal deaths; deaths from epilepsy or aggravation of an existing cardiac disease are classified as indirect.

ICD-10 has been in use in Sweden since 1997, preceded from 1987 to 1996 by ICD-9. Pregnancy-related deaths are all those deaths occurring during pregnancy or within 42 days after the end of pregnancy, irrespective of cause of death. This definition was introduced in ICD-10 to facilitate the identification of maternal deaths under circumstances in which cause of death attribution is inadequate. Fortuitous deaths are defined as deaths from unrelated causes which happened to occur during pregnancy or the puerperium. Along with the reporting method of the British Centre for Maternal and Child Enquiries, we prefer to use the term “coincidental” instead of “fortuitous,” since coincidental is a more accurate description and the term fortuitous could imply “fortunate” 19. Suicides are not included in the current ICD definition of maternal death, in contrast to both the Centre for Maternal and Child Enquiries reports and a proposal for classification by the WHO Working Group on Maternal Mortality and Morbidity Classifications 20. Cancers, accidents, and homicides were classified as coincidental deaths, although the British reports classify hormone-dependent cancers as indirect maternal deaths, and homicides as direct, indirect or coincidental, depending on the individual circumstances b19.

The concept of late maternal death was included in ICD-10 in order to capture deaths from pregnancy-related events that occur between 6 weeks and 1 year postpartum 21. A complication occurring during pregnancy can lead to death more than 42 days later, and increasingly available modern life-sustaining procedures and technologies enable more women to survive beyond this period. For the purpose of international reporting of maternal mortality, only those maternal deaths occurring before the end of the 42-day period are included. However, the recording of late deaths is useful for national analytical purposes.

Different methods have been used to address the problem of underreporting maternal deaths. In a recent WHO publication, an adjustment factor of 1.5 is applied to account for misclassification of maternal deaths in countries whose civil registration is otherwise characterized as complete, that is, with good attribution of cause of death 22. This adjustment factor is the median of underreporting of maternal deaths in civil registration based on available studies. Early pregnancy deaths, later deaths in the postpartum period, deaths at extremes of maternal age (youngest and oldest), and indirect deaths caused by cerebro- and cardiovascular diseases are the most common cases not reported as instances of maternal death 11,23. With the publication of ICD-10, WHO recommended the inclusion on death certificates of questions regarding current pregnancy and pregnancy within 1 year preceding death 18 but this has not been implemented in Sweden, although shown to be useful 24. The benefits of routine linkage of birth and death registers have been shown in several studies 12,13,16,25. In Denmark, in contrast to the other Nordic countries, the number of maternal deaths reported to WHO is based on linkage between the civil registration system and the national patient register 25. Confidential enquiries, which first began in England and Wales more than 50 years ago 19, have identified more maternal deaths compared with the civil registration systems in several countries 9,11,14,25. In Sweden the maternal mortality group of the Swedish Society of Obstetrics and Gynecology was formed in 2007 and has so far assessed five to seven cases yearly, reported on a voluntary basis from Swedish hospitals, but a routine linkage system of registers is not yet in place.

Sweden's official statistics on maternal mortality, reported to the WHO by the National Board of Health and Welfare, is based on the underlying cause of death, defined as the disease or injury that initiated the pathological chain resulting in death; or the circumstances surrounding the accident or act of violence that caused a lethal injury. Thus, only deaths with an underlying cause of death identified in ICD-9 chapter XI (630–676) or ICD-10 chapter XV (O00–O95, O98–O99) or A34 (obstetrical tetanus) are reported as maternal deaths. The thought behind this is to capture deaths directly associated with a pregnancy, whereas death from an aggravated preexisting condition will be assigned the same code as the primary disease. For example, when a woman dies from a myocardial infarction during labor, the underlying cause of death will most likely be coded as cardiac disease (ICD chapter IX), and the pregnancy as a contributing cause of death; she will not be reported as a maternal death, although the definition of a maternal death clearly embraces this indirect death. ICD provides coding rules for assigning an underlying cause of death 18, but the knowledge of clinicians on how to complete death certificates has been shown to be deficient 26. The objective of our study was to use the existing information in Swedish national registers and cause of death certificates to determine the number of maternal deaths that in a way would conform to a greater degree with the ICD-9 and -10 definitions of a maternal death than the method currently used. The “new” number acquired was used to recalculate maternal and pregnancy-related mortality ratios from 1988 to 2007.

## Material and methods

We used three Swedish national registers: the Cause of Death Register (CDR), the Medical Birth Register (MBR), and the National Patient Register (NPR). The study population comprised 27 957 recorded deaths of women of reproductive age (defined by the WHO as 15–49 years old) between 1988 and 2007. These women were identified through the CDR, a record that includes all *residents*, whether that person was a citizen or was present in Sweden at the time of death. However, undocumented migrants and those who died while seeking asylum or visiting Sweden are not included. The register is based on death certificates issued by an attending physician or a physician conducting an autopsy. Underlying and contributory causes of death were coded in the CDR according to ICD-9 from 1988 to 1996, and ICD-10 since 1997. The MBR contains information on women who give birth to a living child, or to a stillbirth with a gestational age ≥28 weeks (since 2008: ≥22 weeks) or a birth weight ≥500 g. The NPR has registered data on hospital discharges since 1964 but it is first considered complete for all public hospitals in Sweden after 1987. Beginning in 2001 the NPR also includes a register of out-patient care. All three records are maintained by the Swedish National Board of Health and Welfare.

Through the CDR we obtained information on deceased women who had an underlying cause of death or a contributory cause of death listed in the ICD chapter “Pregnancy, childbirth and the puerperium” in ICD-9 (630–676) and ICD-10 (O00–O99); obstetric tetanus (A34 in ICD-10); or choriocarcinoma (181 in ICD-9, C58 in ICD-10). By linking CDR to MBR through each resident's personal identification number, we acquired information on women who had given birth within 1 year of their death. When linking CDR to NPR we obtained information on women who had been admitted to hospital or had an out-patient specialist appointment designated with an ICD code that appears in the chapters mentioned above. Using this approach, all women having a diagnosis related to pregnancy in at least one of the three registers within 1 year before they died were identified. Copies of the cause of death certificates of these women were obtained from the National Board of Health and Welfare. The certificates were independently scrutinized by two obstetricians (A.E. and U.H.) and a decision on classifying each as a direct or indirect maternal death, or a coincidental death, was made after discussion. Pregnancy-related mortality was calculated, as well as maternal mortality ratios, both excluding and including suicides, and compared with the 75 cases with an underlying cause of death included in the ICD chapter for Pregnancy, Childbirth, and the Puerperium, that is, maternal deaths reported to WHO by the National Board of Health and Welfare.

SAS version 9.2 and SAS ENTERPRISE GUIDE 4.2 software package (SAS Institute, Cary, NC) were used for the record linkage. Ethics approval for this study was not needed according to Swedish laws on ethical review, since all women were deceased. The Regional Ethics Committee in Uppsala, Sweden, confirmed that the study did not fall into the category of research requiring ethical clearance (2008/381, 14 January 2009).

## Results

**Figure 1:**
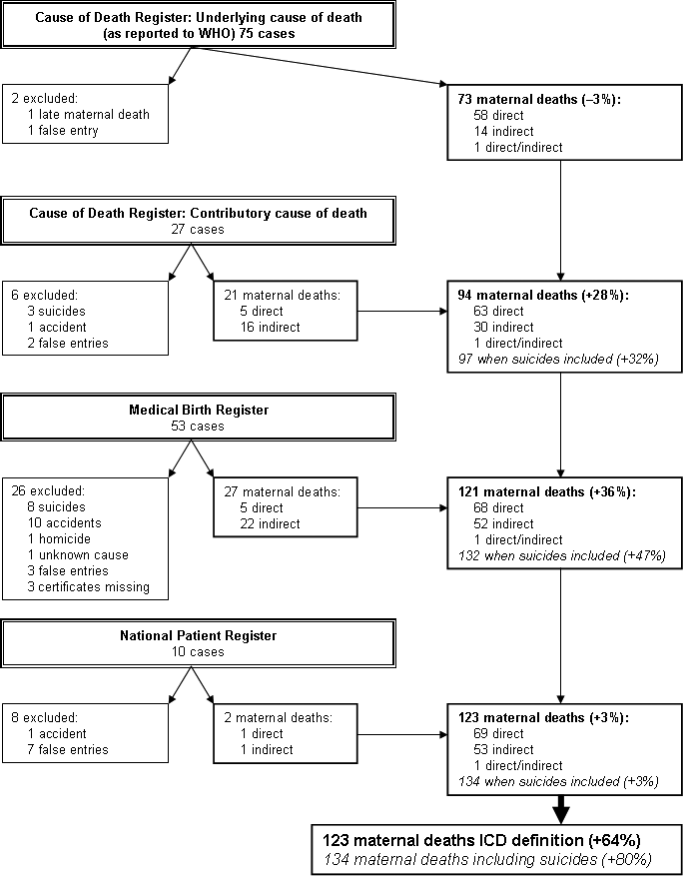
Maternal deaths in Sweden (excluding and including suicides), 1988–2007, identified through Cause of Death Register, Medical Birth Register, and National Patient Register.

In our study population, we identified 491 women in the CDR or NPR with at least one diagnosis from the ICD chapter cited earlier, or a delivery registered in the MBR within 1 year prior to the woman's death. In 164 cases a woman died within 42 days after giving birth or having a pregnancy-related diagnosis in the NPR (Figure 1); 327 women died on day 42 to 364 (Table 1).

**Table 1:** Underlying causes of death in women who died on day 42 to 364 inclusive after termination of pregnancy in Sweden 1988–2007

Cause of death	Number of deaths	Percentage
Malignancy of breast or genital organs	22	6.7
Other malignancy	78	23.9
Disease of the circulatory system	39	11.9
Disease of the respiratory system	11	3.4
Endocrine and metabolic disease	7	2.1
Disease of the nervous system	6	1.8
Sepsis	4	1.2
Other diseases	13	4.0
Pregnancy, childbirth, and the puerperium	2	0.6
Suicide	64	19.6
Traffic accident	31	9.5
Homicide^a^	15	4.6
Other external cause^b^	30	9.2
Unclear cause of death	5	1.5
Total	327	100

a Relationship between victim and perpetrator not coded in the International Classification of Diseases.

b Other accidents, substance abuse, and deaths from external causes by undetermined event.

Of the 75 women with an underlying cause of death connected to pregnancy, one proved to be a late maternal death and one was assessed as a false register entry (discussed below). One maternal death could not be classified as direct or indirect with the information available. When we reviewed cases in the CDR with a contributory causes of death related to pregnancy, another 21 maternal deaths (five direct and 16 indirect) were identified (Figure 1). In these five direct deaths, pregnancy was not noted as the condition precipitating the chain of events that led to the death recorded on the certificate. However, these deaths were correctly coded according to ICD rules. Our assessment was that a complication of the pregnancy was the underlying cause of death. Through the MBR we found 53 deaths listed in the CDR that occurred within 42 days after delivery that were not registered with a diagnosis related to pregnancy. Of those, 27 were assessed as maternal deaths (five direct and 22 indirect). Finally, through the NPR we identified 10 women who died within 42 days after they had been registered in the NPR with a diagnosis related to pregnancy, but without a birth being registered in the MBR or a pregnancy-related diagnosis in the CDR. Two of these were assessed as maternal deaths, one direct (sepsis) and one indirect (epilepsy). In summary, after step-wise register linkage and review of death certificates, we identified 50 “new” highly probable maternal deaths and 11 suicides during pregnancy or the puerperium. There were 2 060 678 live births in Sweden from 1988 to 2007, which gives a maternal mortality ratio of 3.6 if one uses maternal deaths as defined by the underlying cause of death. When we count all 123 maternal deaths, the ratio rises to 6.0, and if we include suicides, the ratio becomes 6.5. The majority of the women in our study population (*n* = 100) were born in Sweden (59 “known” and 41 “new” cases) and 23 were born abroad (14 “known” and 9 “new” cases).

Among the deaths within 42 days after the termination of pregnancy, three cases were excluded as false entries. According to the registers these women had given birth *after* their death, and no cause of death certificates could be found. After reviewing register data and death certificates, we also excluded 10 women we believe were miscoded and had not been pregnant shortly before their death. The most common coding mistake in this group was a diagnosis of postpartum deep venous thrombosis given to a person who died from a malignancy, with no registered birth within a year before her death. Thus, a total of 151 pregnancy-related deaths were identified, giving a pregnancy-related mortality ratio of 7.3/100 000 live births.

Table 1 lists the 327 women who died between day 42 and 364 after delivery or after having a diagnosis related to pregnancy registered in the NPR. Only two women could clearly be classified as late maternal deaths: one peripartum cardiomyopathy and one choriocarcinoma. Both late maternal deaths were identified through the CDR. The cardiomyopathy was coded as the underlying cause of death connected to pregnancy, and thus incorrectly included in the official statistics. In total, four cases of choriocarcinoma were found. Three of those women died after having the disease more than 1 year. No case of obstetric tetanus was found. The ICD-10 diagnoses O96 (death from any obstetric cause occurring more than 42 days but less than 1 year after delivery) and O97 (death from sequelae of direct obstetric causes occurring 1 year or more after delivery) were not assigned to any woman during the survey period.

## Discussion

The major finding of this study is the occurrence of 64% more maternal deaths than those identified through underlying cause of death only, giving a maternal mortality ratio of 6.0 instead of 3.6, which is equivalent to the adjustment factor of 1.5 used by WHO. Nearly all “new” maternal deaths (48 of 50) were found by searching among the contributory causes of death in the CDR and by linking CDR to MBR; only two were identified through linkage with the NPR. Among cases included in the official statistics with an underlying cause of death related to pregnancy complications (that could, therefore, primarily be expected to be a direct maternal death) we identified both direct (58/73) and indirect deaths (14/73). Although assumed to be exclusively indirect deaths, we also found both direct (11/50) and indirect (39/50) maternal deaths among the “new” cases. The same proportion of foreign-born women was found among the cases reported to WHO (14/73) as among the “new” cases (9/50), indicating that immigrants were missed to the same extent as Swedish-born women.

Among the 29 maternal deaths identified through the MFR and NPR (having no pregnancy diagnosis registered in CDR), we assessed 23 cases as maternal deaths (three direct and 20 indirect), based on the combined information from registers and death certificates – despite the fact that there was no information about pregnancy on the certificates. Most probably the doctor completing the death certificate was either unaware of the recent pregnancy, or failed to recognize the importance of recording associations between a death and a pregnancy. Information about the pregnancy was recorded on the death certificate in six cases (three direct and three indirect maternal deaths) but this was not taken into account as an underlying or a contributory cause of death when the death was coded in the CDR. These cases illustrate substandard registration of the cause of death by the presiding doctor, as well as deficient secondary assessment of the death certificate at official level.

We calculated maternal mortality ratios both excluding and including 11 suicides during pregnancy and within 42 days after termination of pregnancy. The Centre for Maternal and Child Enquiries classifies suicides as indirect maternal deaths 19, in contrast to studies strictly applying the ICD-rules 2,25 that classify suicides as coincidental deaths. In the Netherlands, suicides occurring during pregnancy and the puerperium are classified as a maternal deaths only if a clear correlation with the pregnancy can be found; otherwise they are considered coincidental deaths 27. The WHO Working Group on the Classification of Maternal Deaths and Severe Maternal Morbidities has decided to characterize suicides in pregnancy, as well as deaths from puerperal psychosis and postpartum depression, as direct maternal deaths in the coming WHO classification 20. The prevalence of suicides during gestation and the year following the pregnancy has been shown by several studies to be lower than that of suicides of women who were not pregnant 28,29. This protective effect seems to be stronger during pregnancy than postpartum. However, the risk of suicide has been shown to be higher in the year after a miscarriage or an induced abortion than after a term pregnancy 29. Three recent British reports found suicide more common than previously thought and considered it the leading overall cause of maternal death in the United Kingdom 19. Associations between pregnancy and suicide are deserving of further study in Sweden.

The most common underlying causes of death among women dying on day 42 to 364 after their pregnancy were malignancies, suicides, and vascular diseases, just as in the overall population of women of reproductive age 30. Although late indirect maternal deaths may be among these, this could not be verified with the data available. A pregnancy-related psychiatric disorder was mentioned on the death certificates of five women who committed suicide.

By means of record linkage, we have been able to identify deaths that occurred within 1 year of pregnancies recorded in the national registers. Deaths during pregnancy or after a *spontaneous* abortion in women who did not receive specialist care and, therefore, were not registered in the NPR would not be identifiable unless the pregnancy was noted on the death certificate. A death due to complications after an *induced* abortion is reported in the CDR, but induced abortions are not registered in the NPR according to Swedish law. Therefore, other possible deaths within a year after induced abortions (irrespective of cause) could not be identified through the record linkage in this study unless the abortion was noted on the death certificate. No direct deaths due to abortion were registered during this study period or from 1980 to 1988 2. It is unlikely that direct deaths in early pregnancy or after spontaneous or induced abortions would be missed. Women without a complete personal identification number (asylum seekers, undocumented migrants, and visitors) are not included in the CDR, and possible maternal deaths in this group were not identified. Maternal deaths at ages below 15 or above 49 are theoretically possible but were not identified since we started with a population of women who died at a reproductive age according to the WHO definition. Basing our classification on register data and death certificates only, an overestimation of maternal deaths through misclassification could not be ruled out. A careful review of individual medical records might reveal a few coincidental deaths among cases we assessed as indirect maternal deaths, depending on the timing of the death and the medical history of the woman. However, the assessment of medical records was beyond the scope of this study.

Although a maternal death is a rare event in Sweden, surveillance of maternal mortality is a necessity. We need the appropriate tools to detect trends. The analysis of every death by the Swedish Maternal Mortality Group provides a deeper understanding of this unfortunate phenomenon and extracts vital information by scrutinizing the chain of events leading to a maternal death. A Nordic collaboration was formed in 2011 that gives us the opportunity to learn from cases occurring in our five relatively small populations (Denmark, Finland, Iceland, Norway and Sweden) and to return recommendations to health professionals.

The current system for identifying maternal deaths in Sweden according to the ICD definition is inadequate: physicians fail to complete death certificates correctly, information about pregnancy is not always taken into account when certificates are coded, direct maternal deaths are not always coded with an underlying cause of death connected to pregnancy, and indirect maternal deaths are not captured when the underlying cause of death alone is considered. By using information that is already available in national registers and on death certificates, the accuracy of statistical records could be improved substantially. Since maternal death occurs so infrequently, Swedish clinicians may be unaware of the importance of searching for associations between pregnancy and death. The introduction of check boxes on Swedish death certificates for current pregnancy and pregnancy within a year preceding death may increase this awareness. In combination with routine record linkage, it would most probably improve the completeness of maternal death data. International praxis for handling inconsistencies between the ICD definition of maternal death and the ICD rules for coding causes of death also need to be improved to capture maternal deaths better and to increase the comparability of maternal mortality statistics.

## Funding

This work was supported by a grant from the Swedish Council for Working Life and Social Research (FAS 2007–2026) and by the Medical Faculty, Uppsala University.

## Abbreviations

CDR: Cause of Death Register
ICD: International Classification of Diseases
MBR: Medical Birth Register
NPR: National Patient Register
WHO: World Health Organization
